# Antidepressant prescriptions and adherence in primary care in India: Insights from a cluster randomized control trial

**DOI:** 10.1371/journal.pone.0248641

**Published:** 2021-03-19

**Authors:** Aravind Pillai, Katherine M. Keyes, Ezra Susser

**Affiliations:** 1 Department of Epidemiology, Columbia University Mailman School of Public Health, New York, New York, United States of America; 2 New York State Psychiatric Institute, New York, New York, United States of America; Chinese University of Hong Kong, HONG KONG

## Abstract

**Background:**

The World Health Organization recommends that treatment of depression in low and middle-income countries with a scarcity of psychiatrists could be done in primary care and should include prescription of antidepressant medications for moderate and severe depression. Little is known, however, about the actual practices of antidepressant prescription by primary care physicians in low and middle-income countries, nor about adherence by people receiving such prescriptions. In a large study of primary care clinics in Goa, India, we examined the relationship of actual to recommended prescribing practices for depression, among all patients who screened positive for common mental disorder. We also examined other patient and clinic characteristics associated with antidepressant prescription, and self-reported adherence over a one-month period.

**Methods:**

Patients attending 24 primary care clinics were screened for common mental disorders. Those who screened positive were eligible to enroll in a trial to assess the effectiveness of a collaborative stepped care (CSC) intervention for mental disorders. Physicians in the 12 intervention and 12 control clinics (usual care) were free to prescribe antidepressants and follow-up interviews were conducted at 2, 6 and 12 months. Screening results were shared with the physician, but they were blinded to the diagnosis generated later using a standardized diagnostic interview administered by a health counsellor. We categorized these later diagnoses as “moderate/severe depression”, “mild depression or non-depression diagnosis”, and “no diagnosis”. We used a two-level hierarchical logistic regression model to examine diagnostic and other factors associated with antidepressant prescription and one-month adherence.

**Results:**

Overall, about 47% of screened positive patients (n = 1320) received an antidepressant prescription: 60% of those with moderate/severe depression, 48% of those with mild depression or non-depression diagnosis, and 31% with no diagnosis. Women (OR 1.29; 95%CI 1.04–1.60) and older adults (OR 1.80; 95%CI 1.32–2.47) were more likely to receive an antidepressant prescription. While the overall rate of antidepressant prescription was similar in clinics with and without CSC, patients without any diagnosis were more likely to receive a prescription (OR 2.20 95% CI 1.03–4.70) in the usual care clinics. About 47% of patients adhered to antidepressant treatment for one month or more and adherence was significantly better among older adults (OR 3.92; 95% CI 1.70–8.93) and those who received antidepressant as part of the CSC treatment model (OR 6.10 95% CI 3.67–10.14) compared with those attending the usual care clinic.

**Conclusion:**

Antidepressants were widely prescribed following screening in primary care, but prescription patterns were in poor accord with WHO recommendations. The data suggest under-prescription for people with moderate/severe depression; over-prescription for people with mild depression or non-depression diagnoses; and over-prescription for people with no disorders. For all diagnoses adherence was low, especially in usual care clinics. To address these concerns, there is an urgent need to study and develop strategies in primary care practices to limit unnecessary antidepressant prescriptions, target prescription for those patients who clearly benefit, and to improve adherence to antidepressant treatment.

ClinicalTrials.gov Identifier: NCT00446407.

## Introduction

In low and middle-income countries (LMIC), depressive disorders are the second leading cause of years lost due to disability [1]. They are common among primary care patients, but often undetected, and undertreated. As a result, there is a growing call for routine screening and treatment for depression at the primary care level [2], and a collaborative stepped care intervention is now widely supported for this task [3, 4]. Antidepressants are a critical component of this model, and in the World Health Organization (WHO) mental health gap (mhGAP) guidelines for treatment of mental disorders in primary care, antidepressants are recommended for patients with moderate to severe but not mild depression [3].

The mhGAP guidelines accord with evidence from meta-analyses of placebo-controlled trials, which provide robust evidence to support the use of antidepressant drugs in moderate and severe depression [5]. However, people with depressive disorders seen in primary care often have mild depression, less severe and less complicated than depression seen in specialist settings, and spontaneous remission of mild depression is common [6, 7]. The evidence supporting the use of antidepressants for mild depression is equivocal at best. In this context, an exponential increase in the use of antidepressants in primary care in some high-income countries has led to concerns of over prescribing [8–10], potentially due to the misdiagnosis and/or the overestimation of the effectiveness of antidepressants in treating less severe depression [11]. These concerns are even more prominent in LMICs where primary care physicians often have limited training in diagnosis and treatment of mental disorders.

In most LMIC, antidepressants are still more commonly prescribed by mental health specialists and in mental health clinics than in primary care [12, 13]. Recently, under new initiatives, access to antidepressants in primary care is slowly improving in many of these countries. For example, antidepressants, commonly SSRIs, are now available for prescription, free of charge to patients in public primary care clinics in several states in India [14–16]. Yet, little is known about the rate of antidepressant prescriptions, and the symptoms and diagnosis for which they are prescribed in primary care in these countries. The few trials of interventions for common mental disorders in primary care from LMIC were not primarily focused on the use and effect of antidepressants [17–19]. Further, studies from LMIC suggest that adherence with antidepressants, including newer SSRIs is generally poor among primary care patients [20, 21].

Given that the proposed screening for depression in primary care could lead to an increase in antidepressant treatment in LMIC, there is an urgent need to better understand the use of antidepressants following screening in these settings. Therefore, this study examined how the actual practice of antidepressant prescription was related to a standardized research diagnosis to which prescribing physicians were blinded. It was done among people who screened positive for common mental disorders in a large study of primary care clinics in Goa, India. We also examined other patient and clinic characteristics associated with antidepressant prescription. Finally, we analyzed adherence over a one-month period.

## Materials and methods

### Study design and sample

We used data from a cluster randomized control trial, the MANAS trial from Goa, India. The trial was designed to assess the effectiveness of a collaborative stepped care (CSC) intervention in the recovery of primary care patients from common mental disorders (ClinicalTrials.gov Identifier: NCT00446407). The original trial was approved by the Institutional Review Boards of the London School of Hygiene and Tropical Medicine and Sangath, the Indian Council of Medical Research, and an independent Trial Steering Committee. The specific research reported in this article using secondary data from the MANAS trial was designated as exempt (protocol #IRB-AAAQ2151) by Columbia University IRB as there is no interaction with subjects, there is no intervention, and private, identifiable information is not being collected. Details of the trial’s sample and design are described extensively elsewhere, and the summary that follows draws on those descriptions [22].

Adult patients visiting the 24 randomly selected primary health clinics in Goa (12 CSC clinics and 12 usual care clinics) were invited to participate in the mental health screening while waiting to see the physician. The study arms included an equal proportion of public primary care clinics and private general practitioner (GP) clinics. A health counsellor administered the screening questionnaire to those who consented. Only adult patients 18 years or older and expected to be resident in the study communities for the following 12 months were eligible. Patients who screened positive were also evaluated by the health counsellor for any mental disorder during the same visit using the Clinical Interview Schedule- Revised (CIS-R), a structured ICD-10 diagnostic interview schedule used for measurement and diagnosis of non-psychotic psychiatric morbidity. The CIS-R based diagnosis was generated later using a computer algorithm but was not available to the primary care physician during the clinical consultation.

The 12 intervention clinics (CSC clinics) implemented a collaborative care management model with psychoeducation, antidepressants and interpersonal psychotherapy delivered in ‘steps’ based on the severity of the illness and the patients’ response to treatment by a team of providers. While psycho-education was offered to all screened positive participants in CSC clinics, more resource-intensive interventions such as psychotherapy and antidepressants was reserved for participants with moderate to severe distress. In the control clinics (usual care clinics), physicians received screening results, and they could initiate treatments of their choice. Antidepressants were prescribed by physicians in both arms of the trial, while other components of the intervention such as psychoeducation, interpersonal psychotherapy, and collaborative case management were not available in usual care clinics.

**Fig 1:**
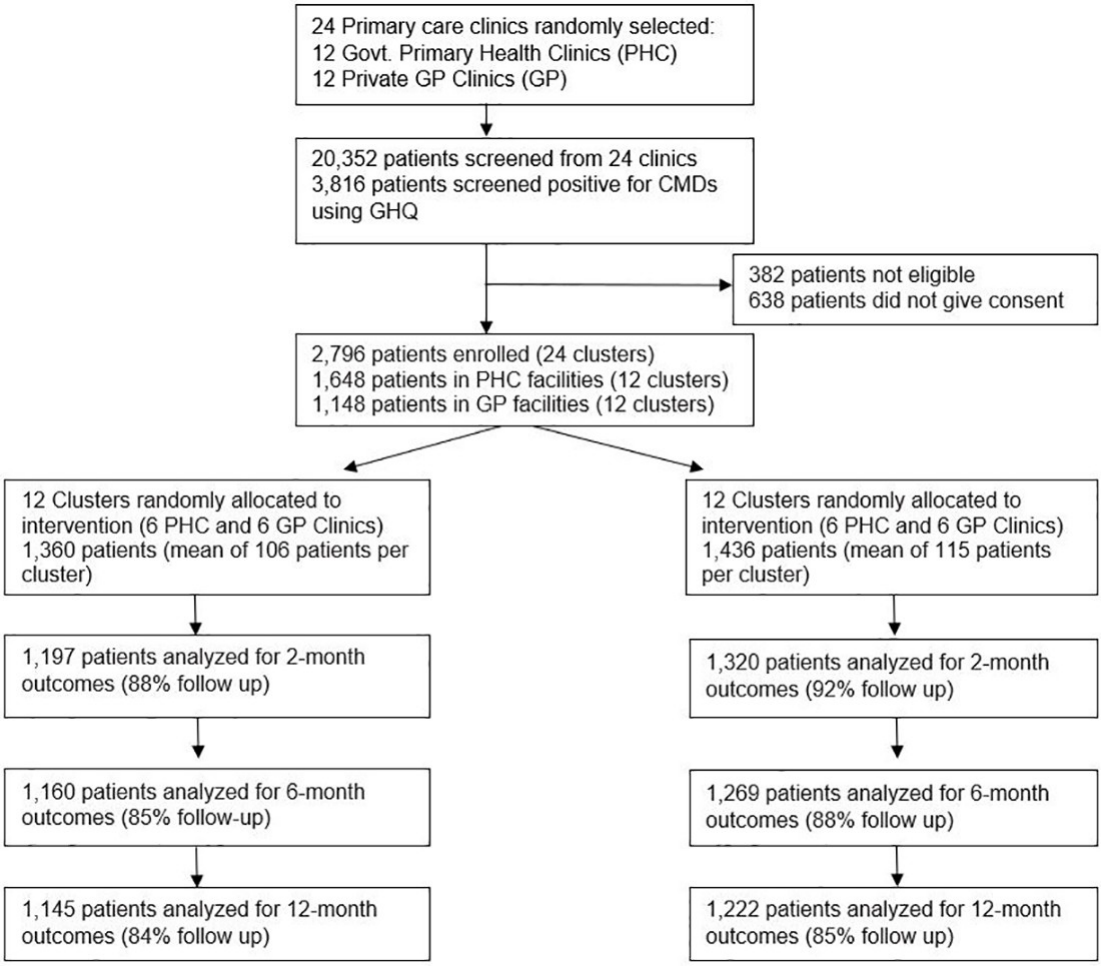
Trial profile.

Fig 1 depicts the progress of participants through the trial. Altogether 20,352 patients were screened, and the number of patients recruited from the collaborative stepped-care clinics and the enhanced usual care clinics were 1360 and 1436 respectively. They were followed up for one year between April, 2007, to September, 2009 and the original study found a modest benefit for the CSC intervention in public primary care clinics, but not in private GP clinics [20, 23].

### Measures

#### The 12-item General Health Questionnaire (GHQ-12)

This study used the GHQ-12 to screen primary care patients for common mental disorders. The GHQ is designed to measure psychological distress in epidemiological studies and population surveys, and to screen for non-psychotic mental disorders in clinics [24]. In our study population, using a cut-off of 5/6 the GHQ-12 showed sensitivity of 73%, specificity of 90%, and a positive predictive value of 61% for a diagnosis of any mental disorder [25].

#### The Clinical Interview Schedule- Revised (CIS-R)

The revised version of the Clinical Interview Schedule is a standardized psychiatric interview that can be administered by interviewers without clinical training in psychiatry [26]. The interview covers 14 symptom domains: depression, depressive ideas, somatic symptoms, fatigue, concentration, worries over physical health, worry, anxiety, sleep, irritability, phobias, panic, compulsions, and obsessions. By applying a diagnostic algorithm to the CIS-R responses, patients could be assigned to six ICD–10 diagnostic categories: generalized anxiety disorder, depressive episode, obsessive–compulsive disorder, phobias, panic and mixed anxiety depressive disorders (MADD) [26]. The diagnosis of MADD is given if a participant has a score of 12 or more on the CIS–R psychological morbidity scale (deemed as the threshold for identifying of clinically significant morbidity) but could not meet criteria for any of the diagnoses generated through ICD–10 diagnostic algorithms [26]. The CIS-R has been extensively used in India, translated and field tested in the study population.

#### Sociodemographic factors

Self-reported age and gender were recorded for each participant during recruitment. Detailed sociodemographic information including ethnicity, marital status and education was compiled during the first follow-up interview within two months of recruitment.

#### Life stressors

Patients were also asked about two major life stressors commonly reported among primary care patients: 1) presence of long-standing physical illness and 2) financial difficulties, i.e. finding it difficult to make ends meet.

#### Clinic level factors (level 2 variables)

The study included only two clinic level variables, namely, the type of clinic (private GP clinic and free public clinic) and the study arm/mental health care model (collaborative stepped care and usual care).

#### Antidepressant adherence

Self-report of antidepressant adherence was recorded during follow-up interviews. Once started, antidepressants were advised for a minimum of 90 days at appropriate dose (minimum 20 mg/day of fluoxetine or a comparable SSRI) In this study we focused on adherence to antidepressants for at least one-month.

### Analysis

We first describe the key characteristics of all screened positive study participants. Next, we report the frequency of antidepressant prescription and self-reported adherence. Then, we examined factors associated with anti-depressant prescription. They included sociodemographic factors, life stressors, and clinical factors. We also analyzed these factors as potential predictors of adherence to one-month of antidepressant use. While the physicians in this study were unaware of the CIS-R based psychiatric diagnosis, they received the results of screening score (GHQ score) categorized as no distress (GHQ score 0–5), mild distress (GHQ score 6–7), and moderate to severe distress (GHQ score >7). We examined antidepressant prescription and adherence by screening score category and the CIS-R diagnosis grouped into following three categories: no diagnosis, moderate-severe depression, and mild depression and other psychiatric diagnosis. Mild depression was combined with other non-depression diagnosis based on data shown that suggested similar severity as well as similar frequency of antidepressant prescription (see S1 Table for distribution of other non-depression diagnoses). Finally, we studied the two clinic level factors, the type of clinic (private GP clinic vs. free public clinic) and the study arm, (CSC vs. usual care) associated with antidepressant prescription and adherence.

The trial data was clustered by clinics with an intraclass correlation coefficient of 0.29 (estimated using a latent variable approach [27]) for the outcome antidepressant prescription. Hence, we used a two-level hierarchical logistic regression model (level one for patients and level two for clinics) with bootstrapped confidence intervals to examine factors associated with primary care physician’s decision to prescribe antidepressants and adherence to antidepressants. The proportion of patients lost to follow-up was 11% at two months, 13% at six months, and 15% at twelve months. Finally, we note that our analyses yield odds ratios, which can be substantially higher than relative risk when the outcome is common, as it is in some of our analyses.

## Results

### Participant characteristics

As described in Table 1, about 82% (n = 2305) of the trial participants were women. This gender distribution is typical of primary care patients in LMIC. Close to half of the participants were 50 years or older and the age distributions for men and women were similar (Gender segregated participant characteristics are shown in S2 Table). Most participants were currently married, but a larger percentage of women were widowed or separated (34% of women and 5% of men P<0.001). Only about one-third of women and half of the men had education above the primary school level (34% vs. 53% P = 0.002). About 46% of patients were poor and finding it difficult to make ends meet. Long standing physical illness including chronic diseases or disability was reported by 47% of patients.

**Table 1:** Sample characteristics, antidepressant prescription and compliance.

	Total % (N)	Antidepressants Prescription % (N)	Adjusted Odds ratio^a^	Antidepressant one-month adherence % ^b^ (N)	Adjusted Odds ratio^1^
**Gender**					
Male	17.6 (491)	41.8 (205)	1	40.1 (84)	1
Female	82.4 (2305)	48.4 (1115)	1.29 (1.04–1.60)	48.6 (542)	1.37 (0.98–1.91)
Total	100 (2796)	47.2 (1320)		47.4 (626)	
**Age**					
18–29 years	10.5 (294)	37.4 (110)	1	30.0 (33)	1
30–39 years	20.4 (571)	42.6 (243)	1.38 (1.01–1.89)	45.7 (111)	2.16 (1.10–4.23)
40–49 years	26.2 (733)	48.0 (352)	1.69 (1.25–2.30)	48.0 (169)	2.68 (1.32–5.45)
50–59 years	19.1 (534)	48.3 (258)	1.56 (1.13–2.15)	48.1 (124)	2.86 (1.49–5.50)
60 years and over	23.8 (664)	53.8 (357)	1.80 (1.32–2.47)	52.9 (189)	3.92 (1.70–8.93)
**Marital Status**					
Single	6.3 (159)	42.1 (67)	1	46.3 (31)	1
married	64.4 (1618)	45.6 (737)	1.06 (0.73–1.54)	47.0 (346)	0.89 (0.46–1.75)
separated/widowed	29.2 (734)	53.0 (389)	1.05 (0.68–1.62)	50.4 (196)	0.83 (0.39–1.75)
**Ethnicity**					
Goan	95.6 (2401)	47.5 (1151)	1	48.3 (556)	1
Migrant	4.3 (109)	37.6 (41)	0.74 (0.48–1.13)	41.5 (17)	0.92 (0.39–2.16)
**Economic situation**					
living comfortably	10.1 (254)	45.3 (115)	1	50.0 (57)	1
just about getting by	44.2 (1107)	47.8 (529)	1.16 (0.85–1.58)	49.5 (262)	0.99 (0.69–1.43)
Difficult to make ends meet	45.7 (1145)	47.9 (548)	1.14 (0.83–1.57)	46.4 (254)	1.16 (0.68–1.96)
**Education**					
Above Primary school	37.0 (929)	45.2 (420)	1	48.1 (202)	1
Primary school or Below	63 (1579)	48.9 (772)	1.10 (0.89–1.35)	48.1 (371)	0.86 (0.64–1.44)
**Long standing physical illness**					
No	52.8 (1280)	44.8 (573)	1	26.0 (149)	1
Yes	47.2 (1145)	50.5 (578)	1.00 (0.83–1.21)	32.9 (190)	1.26 (0.97–1.62)

^a^ Odds ratio adjusted for age and gender.

^b^ Adherence among those who received an antidepressant prescription.

### Antidepressant prescription and adherence

About 47% of screened positive patients (n = 1320) received a prescription for an antidepressant after consulting with the primary care physician. About 5% of participants with a prescription did not start on any antidepressants, while about 47% adhered to the prescription for one-month. Slightly less than one-third of the patients (29%; n = 378) adhered for 90 days or more, the minimum recommended duration for antidepressants in this trial, and 16% reported using antidepressants for 180 days or longer.

### Sociodemographic factors associated with antidepressant prescription and adherence

As depicted in Table 1, women were more likely to receive a prescription for antidepressant than men (42% Vs.48%; OR 1.29 95% CI 1.04–1.60) and prescription receipt increased with age. After adjusting for age and gender, we did not find any clear association between antidepressant prescription and marital status, education, ethnicity, financial situation, or the presence of a long-standing physical illness. We chose a priori not to adjust for symptoms of psychological distress or diagnosis since we considered these variables to be potential mediators and not confounders.

Adherence to antidepressant prescription increased steadily with age (see Table 1). In a multivariable model, patients 60 years and older had nearly four (OR 3.92 95%CI 1.70–9.07) times greater odds of using antidepressants for one-month, compared with those between the age of 18 and 29. While other factors such as gender, marital status, education, ethnicity, long standing physical illness and financial situation did not predict adherence.

### Clinical factors associated with antidepressant prescription and adherence

As described in Table 2, about 55% of screened positive patients (n = 1524) scored in the ‘mild’ GHQ score range for psychological distress and the remaining patients scored in the ‘moderate to severe’ range. Among those patients in the ‘mild’ range 29% received an antidepressant prescription compared with about 70% of patients in the ‘moderate to severe’ range. Of those patients who received antidepressant prescription, 52% in the ‘moderate to severe’ score range adhered to the prescription for one-month, compared with 38% in the mild score range (OR 1.28 95% CI 0.95–1.74).

**Table 2:** Screening score and diagnosis associated with antidepressant prescription and compliance.

	Total % (N)	Antidepressants Prescription % (N)	Adjusted Odds ratio^a^	Antidepressant one-month adherence % ^b^ (N)	Adjusted Odds ratio^a^
**GHQ Case designation**					
mild	54.5 (1524)	28.7 (437)	1	38.4 (168)	1
mod/severe	45.5 (1272)	69.4 (883)	6.54 (5.42–7.9)	51.9 (458)	1.28 (0.95–1.74)
**Any ICD 10 Diagnosis**					
No	19.8 (554)	31.1 (172)	1	42.4 (73)	1
Yes	80.2 (2242)	51.2 (1148)	2.55 (2.03–3.18)	48.2 (553)	1.26 (0.89–1.82)
**Diagnosis**					
No disorder	19.8 (554)	31.1 (172)	1	42.4 (73)	1
Mod-Severe depression	21.1 (589)	59.8 (352)	3.97 (3.01–5.25)	48.0 (169)	1.35 (0.99–1.85)
Other diagnosis	59.1 (1653)	48.2(1653)	2.44 (1.59–3.76)	48.2 (384)	1.22 (0.79–1.89)

^a^ Odds ratio adjusted for age and gender.

^b^ Percentage of adherence among those who received an antidepressant prescription.

Table 2 shows the distribution of psychiatric diagnoses at baseline derived from CIS-R based standardized diagnostic interview categorized into three groups. About 21% (n = 589) of screened positive patients had a diagnosis of moderate to severe depression, 59% (n = 1653) of patients had mild depression or other non-depression diagnosis and 20% (n = 554) did not meet the criteria for any diagnosis. The distribution of diagnoses was similar for men and women. Slightly more than half of the patients (51.5%) who qualified for an ICD-10 diagnosis received an antidepressant prescription and they had 2.55 times greater odds of receiving the prescription than those without a diagnosis. About 60% of patients with a diagnosis of moderate to severe depression received a prescription for an antidepressant and they had approximately 4 times the odds of receiving an antidepressant prescription (OR 3.97; 95%CI 3.01–5.25). On the other hand, 40% of patients with moderate to severe depression (44.2% in usual care clinics & 34.2% in the collaborative care clinics) did not receive any antidepressant prescription, while about 48% of patients with a diagnosis other than moderate to severe depression received a prescription for an antidepressant. Finally, about 31% of screened positive patients who did not qualify for a diagnosis also received a prescription for an antidepressant. We did not find any significant association between specific ICD-10 diagnosis and antidepressant adherence (see Table 2).

While the presence of any of the symptoms of psychological distress increased the odds of receiving a prescription, symptoms of depression (OR 2.00 95%CI 1.67–2.35) and depressive ideas (OR 2.03 95%CI 1.69–2.44) were the strongest factors.

### Clinic related factors (level 2 factors) associated with antidepressant prescription and adherence

As shown in Table 3, 44% of screened positive patients who attended a clinic with CSC treatment model received an antidepressant prescription compared with 50% who attended a usual care clinic. About 38% of screened positive patients who did not meet any diagnosis received an antidepressant prescription in usual care clinic compared with 23% in the collaborative stepped care clinic (OR 2.20; 95% CI 1.01–4.79). For moderate to severe depression, a higher proportion of patients in the collaborative stepped care clinics received an antidepressant prescription while for diagnoses other than moderate to severe depression patients in the usual care clinics were more likely to get a prescription (S3 Table). We also found some variations between antidepressant prescriptions in public primary care and private GP clinics. Overall, patients attending private GP clinics had slightly higher prevalence of receiving (53%; n = 613) an antidepressant prescription compared with public primary care clinics (43%; n = 707), although the confidence interval for this association was imprecise (OR. 1.63; 95%CI 0.77–3.42). Based on the hierarchal model, about 29% (95% CI 17%-43%) of the variance in antidepressant prescription is explained by measured and unmeasured clinic level factors.

**Table 3:** Treatment model and clinic type associated with antidepressant prescription and compliance.

	Total % (N)	Anti-depressants Prescription % (N)	Adjusted Odds ratio^a^	Anti-depressant one-month adherence % ^b^ (N)	Adjusted Odds ratio^1^
**Treatment moded**					
Enhanced usual care	51.4 (1436)	49.9 (717)	1	31.1 (223)	1
Collaborative stepped care (CSC)	48.6 (1360)	44.3 (603)	0.87 (0.40–1.87)	66.8 (403)	6.10 (3.67–10.14)
**Clinic type**					
Public primary care	58.9 (1648)	42.9 (707)	1	43.7 (309)	1
Private GP Clinics	41.1 (1148)	53.4 (613)	1.63 (0.71–3.73)	51.7 (317)	1.00 (0.40–2.49)

^a^ Odds ratio adjusted for gender, age.

^b^ Percentage of adherence among those who received an antidepressant prescription.

Examining the clinic level factors that predicted adherence to antidepressants, we found that about 66.8% of the patients (n = 403) in the CSC clinic used antidepressant for one-month while only about 31% of patients (n = 223) in the usual care model did so (OR 6.10 95% CI 3.67–10.14). Irrespective of their diagnosis, significantly higher proportion of patients in the CSC arm completed the minimum recommended duration (90 days) of antidepressant treatment compared with usual care. However, there was no significant difference in antidepressant adherence between private GP clinics and public primary care clinics. Overall, 43% (95% CI 27%-62%) of the variance in antidepressant use in this study is explained by measured and unmeasured clinic level factors.

## Discussion

This study represents a scenario that would become common if screening for mental disorders becomes a routine practice in primary care clinics and private GP clinics in LMIC. Following screening, the physician recommends an intervention based on the screening result and a brief clinical encounter. Usually, a standardized psychiatric diagnosis is not included in this process, however this study had access to the CIS-R based psychiatric diagnosis that was not available to the physician during the clinical consultation. Our findings are similar to reports from primary care in high income countries that antidepressants are widely prescribed following screening in primary care and a significant proportion prescriptions are given to patients without depression or mild depression that may not indicate pharmacotherapy as per the current treatment guidelines [28]. However, less than half of the patients who received antidepressant prescriptions adhered to their medication for one-month and less than one-third of the patients adhered for 90 days or more, the minimum recommended duration of antidepressant treatment in this study.

Even though physicians in our study were unaware of the CIS-R based psychiatric diagnosis, patients with moderate to severe depression were the most likely diagnostic group to receive an antidepressant prescription, and 60% of them received a prescription. Further 48% of patients with ‘mild depression or other non-depression’ diagnosis also received a prescription. Unfortunately, we do not have enough information to determine the clinical basis for these prescriptions and some of these prescriptions could be appropriate [29]. However, antidepressant prescriptions received by 31% percent of patients with no mental disorder suggest over-prescription following screening in primary care. Although the role of antidepressants in treatment of subthreshold depressive symptoms and mild depression remain unresolved, in general, the latest treatment guidelines do not recommend routine use of antidepressants for mild and sub-threshold depressive symptoms among adults [3, 30, 31]. While physicians sometime prescribe antidepressants for off label indications such as insomnia and pain, there is little evidence that this is ultimately beneficial to patients [28, 32]. On the other hand, about 40% of patients with moderate to severe depression did not receive any antidepressant prescription. Although some of these patients might have received non-pharmaceutical interventions, additional analysis showed poor uptake of key non-pharmacological interventions. While about 96% of screened positive patients in the CSC clinics received psychoeducation at recruitment, less than 2% enrolled in for freely offered interpersonal psychotherapy. Further, in usual care clinics without any non-pharmaceutical treatment options, 45% of patients with moderate to severe depression did not receive any antidepressant prescription compared with 35% without a prescription in the CSC clinics.

On examining factors related to antidepressant prescription, we found slightly higher percentage of prescription for women than men and a steady increase in the prescription with increasing age. Though there is limited information on antidepressant prescription in relation to gender and age distribution from LMIC, a recent study comparing antidepressant use in five European countries reported a similar increase in prescription with increasing age and among women [33]. In our study population the prevalence of depression was similar across age bands, but those in the older age groups were still more likely to receive a prescription. This is a reason for concern as older patients are more sensitive to adverse effects of antidepressant medications and drug interactions, as they frequently have comorbidities and multiple medications [34, 35].

While nearly half of the screened positive patients received an antidepressant prescription, adherence to antidepressant was relatively poor. About 36% of patients with a prescription did not start the antidepressant or discontinued within 15 days. The percent of patients who completed one-month and six-months of antidepressants were 47% and 16% respectively. While little is known about antidepressant adherence in primary care from LMIC, a recent community based study from India reports better adherence with 45% patients completing 12 weeks and 33% of patients completing 24 weeks of antidepressant treatment [36]. In that study, prescriptions were based on psychiatric diagnosis using a structured psychiatric interview, and only patients with moderate to severe illness were included, and they were followed up at home every four weeks. Targeting antidepressants to patients with appropriate diagnosis, moderate to severe illness, and regular follow-up could be possible reasons for the better adherence reported. Meanwhile, a review of antidepressant adherence that combined data from 8 studies from high income countries found an averaged adherence of about 53% at 6 months among primary care patients [37]. Thus, although antidepressants were widely prescribed following screening in our study clinics, adherence to antidepressants was relatively poor compared with high income countries.

Systematic reviews have highlighted inconsistent associations between commonly studied sociodemographic factors and adherence to antidepressants [38, 39]. Nevertheless, among the sociodemographic factors, older age appears to be consistently related to adherence and we found that those under thirty are the most likely to be non-adherent, similar to a recent study from the US that reports greater odds of antidepressant self-discontinuation associated with younger age (18–30 years), with a gradient along the age spectrum [40]. While some studies suggest that more vulnerable patients are less adherent to medication, we did not find association between antidepressant adherence and factors such as education, financial situation, and migrant status.

In general, studies examining clinical predictors of antidepressant adherence including diagnostic subtypes, comorbidities, etc., have remained inconclusive [39]. Examining some of these factors, we found that patients with moderate- severe depression were slightly more likely to adhere with antidepressants for one-month although the relationship was not statistically significant at 95% confidence level (OR 1.35 95%CI 0.99–1.85).

A key element of this study is the collaborative stepped care program for mental disorders implemented in half of the study clinics. We expected lower rates of antidepressant prescription in these clinics due to availability of non-pharmacological treatment options, yet, antidepressant prescriptions rates in these clinics were not appreciably different from those clinics without these treatment options. A plausible reason for the high rates antidepressant prescription in these clinics, could be due to the low demand for interpersonal psychotherapy as described earlier. Interpersonal psychotherapy required patients to visit the clinic at regular intervals, and a qualitative study based on this trial reported that patients were reluctant to return to clinic for regular follow-ups due to a number of reasons [41]. On the other hand, we found significantly better adherence to antidepressants among patients attending the CSC clinics. On possible reason for this finding could be psychoeducation received by 96% of screened positive patient in the CSC clinic. Psychoeducation clarified the nature of their emotional distress and symptoms, and the importance of treatments.

### Limitations

We acknowledge some of the limitations of this study. First, the lack of information on screened negative patients limit us from a full understanding of antidepressant prescriptions in primary care. Second, about 11%, 13% and 15% of patients were lost to follow-up at two months, six months, and twelve months, respectively. Those lost to follow-up were more likely to be younger and male, and to the extent that these factors are associated with the outcome, the effect estimate may be impacted. Third, in few clinics, services were provided by more than one physician and a three-level hierarchical analysis would have been more appropriate to account for providers clustered in clinics, unfortunately provider level data was not collected in the original trial. We also have limited information on physician related factors such as training, years of experience, physicians’ diagnosis of the patients, and the decision-making process that contributed to prescribing to draw definite conclusions on the appropriateness of antidepressant. Fourth, the interpretation of the results is somewhat vulnerable to the validity of the diagnosis based on the revised Clinical Interview Schedule (CIS-R). Finally, self-reported adherence to antidepressants is prone to recall bias as well as social desirability bias. Despite these limitations, to the best of our knowledge this is the first study that focuses on antidepressant prescriptions following screening in primary care in a low or middle-income country.

## Conclusion

In this study on treatment for common mental disorders in primary care from a middle-income country, we found that antidepressants are widely prescribed following screening. However, adherence to antidepressants is poor compared with high income countries. We also found a potential incongruity between antidepressant prescription decision and clinical diagnosis, leading to under-prescription for conditions such as moderate to severe depression and over-prescription for less severe disorders. While many patients with moderate to severe depression could benefit from antidepressants, a significant proportion of patients with less severe disorders also received anti-depressant prescription despite the availability of non-pharmacological treatment options. In some of these less severe cases providing antidepressants could be counterproductive, hampering patients from finding non-pharmacological solutions [32]. Further, when patients are prescribed antidepressants in the absence of proven indications, they are still subject to potential side effects and adverse events, along with the unnecessary cost [28]. To address these concerns, there is an urgent need to study and develop strategies in primary care practices to limit unnecessary antidepressant prescriptions, target prescription for those patients who clearly benefit, and to improve adherence to antidepressant treatment.

## Supporting information

S1 Table Distribution of diagnoses and antidepressant prescription. (DOCX)

S2 Table Characteristics of the study participants by gender. (DOCX)

S3 Table Differences in antidepressant prescription by types of clinics and diagnosis. (DOCX)
