# Supplementary material for: Antidepressant prescriptions and adherence in primary care in India: Insights from a cluster randomized control trial
Source: PLoS One. 2021 Mar 19;16(3):e0248641. doi: 10.1371/journal.pone.0248641 (PMC7978355; doi:10.1371/journal.pone.0248641)
Supplement: S1 Table — (DOCX) [file pone.0248641.s001.docx]

**S1 Table. Distribution of diagnoses and antidepressant prescription**

| **Diagnosis** | **Total % (N)** | **Antidepressants**  **Prescription % (N)** |
| --- | --- | --- |
| No disorder | 19.8 (554) | 31.1 (172) |
| Mod-Severe depression | 21.1 (589) | 59.8 (352) |
| Panic | 4.8 (134) | 49.3 (66) |
| Mild Depression | 6.3 (177) | 46.3 (82) |
| Phobias | 5.2 (146) | 52.7 (77) |
| Generalized anxiety | 5.9 (164) | 50.0 (82) |
| Mixed anxiety depressive | 36.9 (1032) | 47.4 (489) |
