# Supplementary material for: Antidepressant prescriptions and adherence in primary care in India: Insights from a cluster randomized control trial
Source: PLoS One. 2021 Mar 19;16(3):e0248641. doi: 10.1371/journal.pone.0248641 (PMC7978355; doi:10.1371/journal.pone.0248641)
Supplement: S2 Table — (DOCX) [file pone.0248641.s002.docx]

**S2 Table. Characteristics of the study participants by gender**

|  | **All participants**  (n=2796) | **Women**  n=2305 (82%) | **Men**  n=491 (18%) | **P value*** |
| --- | --- | --- | --- | --- |
|  | N (%) | N (%) | N (%) |  |
| **Age** *(n=2796)*  18-29  30-39  40-49  50-59  60 & above | 294 (10.5)  571 (20.4)  733 (26.2)  534 (19.1)  664 (23.8) | 239 (10.4)  466 (20.2)  628 (27.3)  438 (19)  534 (23.2) | 55 (11.2)  105 (21.4)  105 (21.4)  96 (19.6)  130 (26.5) | P=0.840 |
| **Marital Status** *(n=2511)*  Never married  Married  Widowed/separated | 159 (6.3)  1618 (64.4)  734 (29.2) | 100 (4.8)  1278 (61.1)  725 (34.2) | 59 (14.1)  340 (81.3)  19 (4.6) | P<0.001 |
| **Ethnic group** *(n=2510)*  Goan  Migrant | 2401 (95.7)  109 (4.3) | 2013 (96.2)  79 (3.8) | 388 (92.8)  30 (7.2) | P=0.172 |
| **Education** *(n=2508)*  Above primary school  Primary school or below | 929 (37.0)  1579 (63.0) | 710 (34.0)  1381 (66.0) | 219 (52.5)  198 (47.5) | P=0.002 |
| **Financial situation** *(n=2506)*  Comfortable or Just about getting by  Finding it difficult | 1361 (54.3)  1145 (45.7) | 1105 (52.9)  984 (47.1) | 256 (61.4)  161 (38.6) | P=0.163 |
| **Long standing physical illness/ disability** *(n=2425)*  No  Yes | 1280 (52.8)  1145 (47.2) | 1066 (52.6)  961 (47.4) | 214 (53.8)  184 (46.2) | P=0.844 |

*Derived from cluster adjusted Chi square test
