# Supplementary material for: Antidepressant prescriptions and adherence in primary care in India: Insights from a cluster randomized control trial
Source: PLoS One. 2021 Mar 19;16(3):e0248641. doi: 10.1371/journal.pone.0248641 (PMC7978355; doi:10.1371/journal.pone.0248641)
Supplement: S3 Table — (DOCX) [file pone.0248641.s003.docx]

**S3 Table. Differences in antidepressant prescription by types of clinics and diagnosis**

| Diagnosis | Usual Care | Collaborative stepped care^a^ | Adjusted OR^2^ |
| --- | --- | --- | --- |
| **No diagnosis** | 38.4 (112) | 22.9 (60) | 2.20 (1.03-4.70) |
| **Mod-Severe** | 55.8 (198) | 65.8 (154) | 0.62 (0.34-1.14) |
| **Other diagnosis** | 51.6 (407) | 45.0 (389) | 1.19 (0.51-2.80) |

1. Reference group

2. Odds ratio adjusted for gender and age
